# Dermatology and Syphilis

**Published:** 1876-07

**Authors:** 


### V. DERMATOLOGY AND SYPHILIS.

1. *Syphilitic Headache and Neuralgia Cured by small and repeated Doses of Calomel.* PETER. (*Lancet*, July, 1875.)

The author gives the details of several cases treated in the manner recommended by Trousseau, viz.: giving about one-sixtieth of a grain of calomel every hour. The interesting features of this methed of medication, as described by Dr. Peter, are: 1, The rapidity of its action; 2, The fact of its success in cases where the really specific treatment of yphilis fails. It constitutes, in a manner, *the* medicine of nocturnal syphilitic pain, but cannot replace the other plan of treatment for other syphilitic manifestations. Its use is indicated whenever the pain is intense and induces asomnia. It diminishes pain and its consequences the very first night it is given, and generally extinguishes suffering by the second night. The treatment may be carried on for three days, and that period of time is almost always enough for its success. If, however, the desired result has not been obtained, it ought then to be suspended for one or two days, so as to prevent salivation, and it can then be resumed afterwards for two days successively, in which case Dr. Peter has never seen it fail.

The plan of treatment is thought to be efficacious, because: 1st, The drug is mercury; and, 2d, The absorption of these very small doses is exceedingly rapid, and the repetition of the action takes place every hour. Whatever the action may be, adds Dr. Peter, it is to Trousseau that we are indebted for the idea of using calomel in this manner, and to him belongs all the credit.

2. *Herpes Zoster Gangrænosus Recidivus.* PROF. KAPOSI. (*Wiener Med. Wochenschr.*, 1876, No. 1.)

At a meeting of the Vienna Society of Physicians, in November, Prof. K. exhibited a unique case, a patient who has had an eruption of herpes in the course of the same nerves (cervico-brachial plexus) five times within eighteen months, and whose herpes always assumed a gangrenous character. The first eruption (right forearm, shoulder and sternum) was observed in April, 1874; the second (the same as the first) in June, 1874; the third (forearm and lower part of arm) in January, 1875; the fourth (like the third) in June, 1875; and the fifth in October, 1875. On October 17, the patient, a woman, aged 44 years, experienced sharp pains in the right arm and shoulder; on the 19th, the herpetic eruption began, and on the 20th already extensive gangrenous patches existed. October 21, Dr. K. found a greenish-black eschar, with ragged outlines, about the size of a silver dollar, on the right shoulder, and a number of smaller brownish eschars in its vicinity. The spinous processes of the seventh cervical, and of the dorsal and lumbar vertebræ, were painful and very tender to the touch. Oct. 22, violent pain in the right arm and shoulder; at the upper border of the scapula were two red and swollen patches of the size of a silver dollar, with numerous vesicles, isolated and grouped together, and minute eschars. Oct. 25, the vesieles are drying up. Oct. 28, to the right side of the spinous process of the second dorsal vertebra a small group of vesicles, from which a red stripe, two inches and one-half in length, and one inch in width, extends upward beyond the border of the scapula. Upon that stripe numerous vesicles appeared, which, on Oct. 31, were again in a state of desiccation; on the same day an herpetic eruption in the region of the sixth rib. In all eruptions the great tendency to gangrene was noticeable.

3. *Psoriasis of the Tongue.* TRÉLAT. (*Jour. de Med. et de Chir.*, Jan., 1876.)

The connection between lingual psoriasis and epithelioma, is illustrated in three cases. A patient with his tongue covered by psoriasis was operated upon one year later for lingual epithelioma, and died soon afterward. A second patient had at the same time epithelioma of the border of the tongue and extensive psoriasis upon the other surfaces of the organ. Ablation was not followed by its return. In a third case, a man affected with lingual psoriasis for six years had removed from his tongue a vegetating growth having the microscopic characters of epithelioma. This does not go to show that the diseases are identical, but that both may be produced by similar irritation.

4. *Alteration of the Colors of the Hair and Skin after Scarlatina.* DR. WALLENBERG. (*Vierteljahresschr. f. Dermatol. und Syphilis*, III, 1.)

H. W., aged 21 years, had a violent attack of scarlet fever, which was remarkable for the copious serous exudations in the skin. Almost over the whole body the epidermis was raised from the cutis, as though large blisters had been applied. The epidermis was thrown off in large flakes, and the exposed Malpighian layer healed over slowly with a new epidermis. The patient lost all the hair of the scalp, beard, genitals, eyebrows, eyelashes, and the nails of his fingers and toes. But the most remarkable feature of this case was, that the patient, who had a dark complexion and dark brown hair before his sickness, exhibited, after the scarlet fever, the whitest hair and the fairest complexion that were ever observed in an albino. His skin, also, had become exceedingly sensitive, so that the irritation of mercurial ointment, or exposure to the direct sunlight, repeatedly occasioned eczema with subsequent cutaneous desquamation.

5. *Anatomy of Lupus Erythematodes.* PROF. ED. GEBER. (*Vierteljahresschr. f. Dermatol. und Syphilis*, III, 1.)

Pieces of lupous skin, taken either from living or dead bodies, were examined at once or hardened in a solution of chromic acid and diluted alcohol. The slides were colored by carmine or Leonard's ink, and then put in glycerine. The professor gives a detailed description, illustrated by lithographs, of the anatomical changes of the integument during the incipient, progressive and retrogressive stages of lupus. The substance of this report may be given as follows: The morbid process begins with a dilatation of the capillaries in the cutaneous papillæ; the cells constituting the walls of these distended capillaries become enlarged, and project into the lumen of the vessels, thus disturbing the smoothness of their walls. The current of the blood is diminished, and the blood cells accumulate until they densely fill the capillaries, when the leucocytes begin to emigrate through the walls of the vessels. In the next stage, therefore, clusters and heaps of lymphoid cells are found to surround the capillaries and small vessels, and to penetrate with them into the neighboring connective tissue of the cutis; and also to invest and crowd into the follicular structures of the skin. These products of proliferation, however, subsequently undergo a fatty degeneration, and are reabsorbed. According to the duraation and intensity of the disease, the affected cutis exhibits the appearance of a more or less atrophic state.

The result of Prof. G.'s researches agrees very perfectly with what Dr. George Thin said of the histology of lupus erythematodes (*London Lancet*, Jan., 1875): "There was enormous dilatation of the capillaries, which was most marked in the papillæ and around the sweat glands, the contour of the capillaries being mostly indicated by red blood corpuscles, with which they were filled, but the vessels themselves being visible in some of the sections. * * The fact that this condition of the capillaries was found in such an early stage of the disease, and before any other changes, and that it could, if persistent for any length of time, give rise to all the changes described by other observers, led me to doubt whether lupus erythematosus *primarily* affects the glands of the skin."

6. *Rheumatismal Bullous Erythema with Aphthous Fever.* FÉRÉOL. (*La France Médic.*, Dec. 20, 1875.)

A woman fifty-two years old had been subject to intense neuralgic pain accompanying the menstrual epochs, and at twenty-six suffered from severe acute articular rheumatism, with cardiac complications. Great difficulty in motion had resulted and lasted for two years. A suppurating peri-uterine phlegmon opened into the rectum at thirty. A deep abscess of the thigh and possibly of the iliac fossa, consecutive to a contusion, had also occurred. A brick dust deposit in the urine, when noticed, was always accompanied by better general health. The menopause occurred normally at fiftieth year, with some hæmorrhoidal tumors. Some sweating and itching of the skin had been noticed. Appetite, digestion and stools normal. Father and mother were rheumatic.

On Aug. 15 there were slight traces of buccal aphthæ without ptyalism. A bimanual eruption was found, insupportably painful, producing insomnia and a cerebral excitation akin to delirium.

From the points of the fingers to a short distance above the radio-carpal articulations, the skin was uniformly of a vinous red color, as if stained with raspberry juice. The line of demarkation was distinct and brusque at the wrists without any elevation of the skin. The color faded to a yellowish shade under pressure, never completely disappearing. Over the fingers the skin was tense and smooth, as if stretched over wood, with obliteration of the natural folds, but no œdema. Over the hand it was also non-œdematous, but swollen, as if infiltrated with a dense liquid not easily displaced by pressure—the latter requiring to be made with gentleness, on account of the severe pain it elicited. Over this uniformly red surface were three or four points on each hand, where pemphigoid bullæ appeared—irregular and elongated, but of moderate dimensions.

